# Skin Prick Test with Self-Saliva in Patients with Oral Aphthoses: A New Diagnostic Pathergy for Behcet’s Disease and Recurrent Aphthosis

**DOI:** 10.2174/187152811795564109

**Published:** 2011-06

**Authors:** Ari Togashi, Sanae Saito, Fumio Kaneko, Koichiro Nakamura, Noritaka Oyama

**Affiliations:** 1 Institute of Dermato-Immunology and Allergy, Southern TOHOKU Research Institute for Neuroscience, Koriyama, Fukushima 963-8563, Japan; 2 Department of Dermatology, Saitama Medical University, Saitama, Japan; 3 Department of Dermatology, Fukushima Medical University School of Medicine, Fukushima, Japan

**Keywords:** Aphthous ulceration, Behcet’s disease, pathergy reaction, prick test, recurrent oral aphthosis, saliva.

## Abstract

There may be some difficulties to differentiate Behcet’s disease (BD), recurrent aphthosis (RA), and herpetic aphthous ulceration, from other mimicking oral disorders. Despite of unexpected sensitivity and responsiveness, the skin pathergy test regarding a non-specific hypersensitivity has long been thought as one of auxiliary diagnostic benefits for BD.

To determine the potential usefulness and disease specificity of the prick reaction with saliva, a skin prick test with neat and filter-sterilized saliva was performed on the forearm skin of 26 individuals; 10 patients with BD (8 incomplete type without uveitis, 1 complete type, and 1 neurological type), 5 with RA, 3 with herpetic oral aphthosis, 2 with erythema nodosum alone, and 6 healthy controls. We assessed the skin reaction at 48 hours after pricking, and the pricked skin lesions were biopsied and analyzed immunohistologically.

Nine of 10 BD patients (90 %) exhibited an indurative erythema at the skin site pricked with self-saliva, whereas 3 of 5 RA patients (60%) were relatively weak reaction. Pricking with filter-sterilized saliva failed to recapitulate any of positive skin reactions, albeit a faint erythematous dot appeared in a few BD patients, implicating the involvement of causative microorganism(s) in oral bacterial flora. Culture of saliva from 3 randomly chosen BD patients revealed numerous streptococcal colonies on Mitis-Salivarius agar. Histology of the pricked skin sites showed perivasucular inflammatory infiltrates, composed of CD4+ T cells and CD68+ monocyte/macrophage lineage, a feature consistent with a delayed type hypersensitive reaction.

Our results suggested that skin prick test using self-saliva (a new diagnostic pathergy) can be a simple and valuable *in vivo* diagnostic approach for differentiating BD and RA from other mimicking mucocutaneous diseases. The positive skin prick may be triggered by resident intra-oral microflora, particularly streptococci, and may in part address the underlying immunopathology in BD.

## INTRODUCTION

Although oral aphthous ulceration can be seen in a variety of mucocutaneous diseases, there may be some difficulties to characterize recurrent aphthosis (RA), the early clinical stage of Behcet’s disease (BD), and herpetic aphthous ulceration [1, 2]. The oral lesions in these 3 diseases often display the similar ulcerative shape in appearance, but RA and herpetic aphthosis do not develop any of systemic symptoms. BD is a chronic multisystem inflammatory condition characterized by recurrent manifestations in mucocutaneous [e.g. oral and genital ulceration, erythema nodosum (EN)-like eruption, acneic eruption, thrombophlebitis], ocular, and vascular area, and relatively lesser with digestive tract and/or nervous system. Among these variable clinical symptoms, oral aphthous ulceration may represent the initial signature of BD. Besides, there may be considerable variety and concomitant occurrence of these multiple symptoms, resulting in the potential difficulty for conclusive diagnosis of the disease. Although the etiology of BD remains unclear, the pathogenic axis has gradually closed to two distinct scenarios, genetic background and extrinsic triggering factors. The former genetics firmly include the higher association (~60%) with specific HLA haplotype, HLA-B51, without regard to their clinical phenotypes [3-8]. In contrast, the oral unhygienic condition has been suspected as one of the extrinsic factors, because periodontitis, decayed teeth, and chronic tonsillitis are frequently observed in the oral cavity of BD patients, and also, the exacerbation of these orodental conditions has a close association with the disease activity of BD [9-14]. A series of our investigation relevant to the intra-oral circumstances have suggested that more than one-third of BD patients tend to acquire a delayed type hypersensitivity (DTH) against certain streptococcal strains, as assessed by the skin prick test with purified streptococcal antigens [9, 10, 15-17]. Based on the diagnostic criteria established by International Study Group for BD [18], this so-called “pathergy reaction” has been categorized as a minor reference in the renewaled Japanese diagnostic criteria for BD in 2003 [19, 20]. However, patients with RA may also show the hypersensitivity to streptococci [17], and moreover, the standardized streptococcal antigens for diagnostic purpose of BD is currently difficult to prepare. In this study, we aimed to determine the potential benefit and reliability of skin prick test using self-saliva, which mostly contains streptococci, for *in vivo* diagnosis of BD, and if any, to differentiate this from other mimicking mucocutaneous disorders.

## MATERIALS AND METHODS

### Patients

Patients enrolled in this study have attended to the Dermatology Clinic, Southern TOHOKU General Hospital. All 10 patients with BD were diagnosed by the criteria based on the International Research Group for BD and they were clinically classified by the Japanese criteria [6, 20]; 8 incomplete type without uveitis (3 males and 5 females; mean ages, 33 year-old), a complete type with uveitis (23 year-old male) and a neurologic type (55 year-old male). Comparative controls include 5 patients with RA (mean age, 28 year-old), 3 females with herpetic oral aphthosis, 2 females with EN alone (mean age, 50 year-old), and 6 healthy subjects (2 males and 4 females; mean age 40 year-old). All patients approved to receive the skin prick test for the diagnostic purpose after the precise informed consent. Except 1 BD patient (case #10, Table **1**), systemic medication, including corticosteroids, immunosupressants, colchicines, and anticoagulants, was discontinued 2 week before the skin prick. These clinical details have been recognized by the local ethical committee.

### Detection of Streptococci in Saliva

Saliva from patients and control subjects was obtained by sterilized swabbing cotton, and directly cultured on Mitis-Salivarius (MS) agar with 1% tellunite solution (Difco Lab., Detroit, USA). This agar allows growing up streptococcal species alone [21].

### Skin Prick Test

Skin prick test was performed according to our previous study with minor modification. Part of freshly obtained self-saliva was sterilized by a syringe filter with 0.2 µm pore membrane (Nalgene, NY), and immediately used for the following prick study. The forearm skin of the patients and control subjects was pricked with either neat or filter-sterilized self-saliva (FS self-saliva) by using a Prick-Lancetter (OY ALGO AB Espoo/Esbo Puh90-50991, Sweden). The skin reaction was measured in diameter 48 hours after the skin prick. Based on our preliminary data and the original pathergy reaction mostly seen in BD patients [18, 22], we recognized the following 3 skin reactions as “positive”: i) erythema and/or induration more than 10 mm in BD patients, ii) those more than 5 mm in other diseased and control subjects, iii) pustules more than 2 mm 24 hours in BD patients.

### Histochemistry

To characterize the profile of inflammatory infiltrates at the pricked skin sites, a skin biopsy was obtained from representative erythematous reaction in 3 distinct BD patients. The infiltrating cells were immuno-stained with anti-CD3, CD4, CD5, CD8, CD20, CD56 and CD68 monoclonal antibodies (DAKO), and then with HRP-conjugated secondary antibodies. Signals were detected by diaminobendizine/H2O2 substrates. The positive cells were counted under x200 high power magnification of a light microscope at least 3 distinct fields, and then expressed as mean cell numbers.

## RESULTS

### Strepcotoccal Species were Detectable in the Culture from BD Patients’ Saliva

**Fig. (1):**
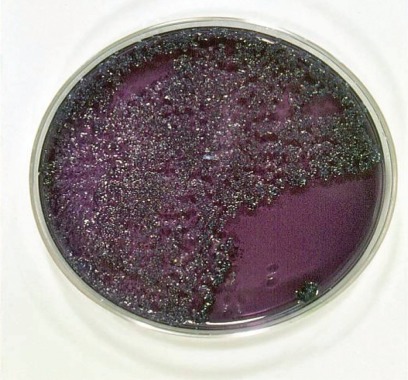
Streptococcal colonies were grown from self-saliva of a representative BD patient in MS agar dish. Numerous, but three major colonies of *Streptococci* appeared at 5th day culture (**A**). No colonies were found in the area of filter-sterilized salvia (**B**).

Although Streptococcal species normally exist in oral micro-flora, certain streptococcal species, *Streptococcus (S) sanguinis* and *S. mitis,* were increased in saliva of BD patients [13, 14]. Saliva culture from 3 randomly chosen BD patients and healthy controls demonstrated at least 3 major colonies of Streptococcal strains at the 3^rd^ to 5^th^ days in MS agar dish (Fig. **1A**). After filter-sterilization, culture from the same patients’ saliva did not show colony formations (Fig. **1B**).

### Patients with BD and RA Showed Positive Pathergy Reaction with Self-Saliva Prick

**Fig. (2):**
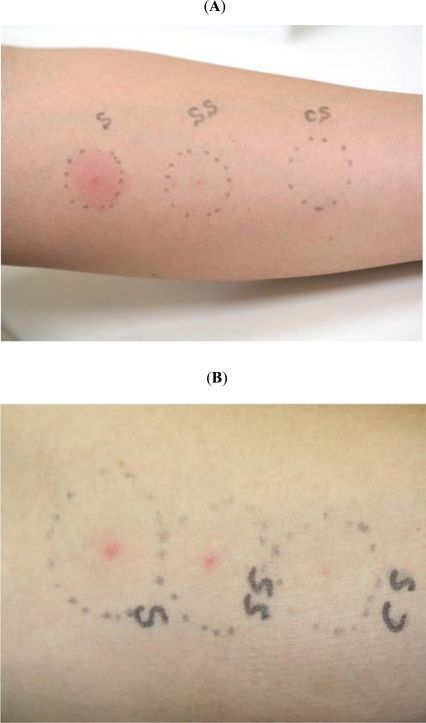
Skin prick reactions by freshly obtained self-saliva, filter-sterilized self-saliva, and control saline. (**A**) In a 27 year-old male BD patient with active aphthous ulceration (case #4, Table **1**), an intense and indurative erythema with a small pustule was established by a skin prick with neat self-saliva (S, left), but showed a tiny dot reaction by filter-sterilized self-saliva (SS, middle). A skin prick with control saline did not cause any of skin reactions (CS, right). (**B**) In a 26 year-old female BD patient without aphthous ulceration (case #3, Table **1**), a very mild erythema but central pustules were established by a skin prick with neat (S, left) and filter-sterilized self-saliva (SS, middle), and even by control saline (CS, right).

In 9 of 10 BD patients (90 %), skin prick with neat self-saliva induced indurative erythema more than 10 mm (Table **1**). Five of the 9 positive patients also showed a tiny pustule at the pricked skin site. Interestingly, four of 7 positive patients turned into negative by filter-sterilized (FS)-saliva skin prick (Fig. **2B**), and the remaining 3 patients, who had active aphthous ulceration, exhibited a pustular formation alone (Fig. **2A**). A young female BD patent, who treated with a low dose of oral corticosteroid, showed a very mild erythema without pustules (case #10, Table **1**).

In disease controls, three of 5 RA patients (60%) had a weak erythema with self-saliva skin prick, but they showed negative reaction with FS-saliva skin prick (Table **1**). Contrary to these, patients with EN alone and herpetic oral aphthosis, as well as 6 healthy volunteers, did not show any of positive reaction with neat and FS self-saliva (Table **1**).

### Inflammatory Infiltrates at the Pricked Skin Sites Revealed Th1-Dominant Immune Response

**Fig. (3):**
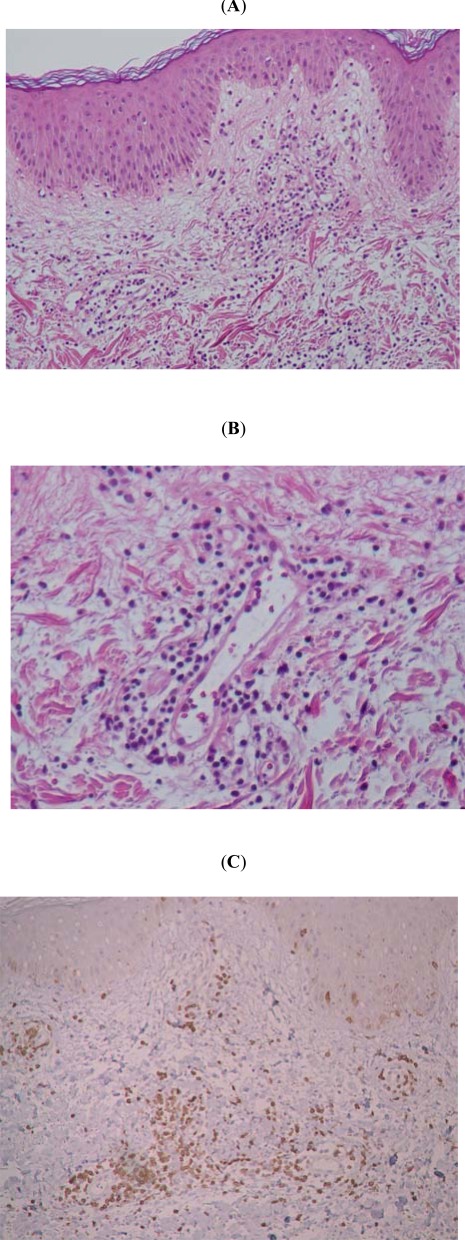
Histological and immunohistological characteristics of the pricked skin sites. Histology revealed interstitial edema and intense lympho-histiocytic infiltration (**A**, x40), but not a typical feature of vasculitis (**B**, x200), in the upper dermis. These inflammatory infiltrates are mainly composed of CD4+ T cells (73.4%) and to lesser with CD8+ T cells (12%) and CD68+ cells (38%) (**C**). No other cell sources, such as CD20+ or CD56+ cells were found.

We next tried to characterize the profile of inflammatory infiltrates at the pricked skin sites in BD. Biopsy specimens from the pricked skin in 3 randomly chosen BD patients were immuno-stained with paneled antibodies. Basically, routine histological examination of all sections from 3 distinct BD patients’ skin revealed interstitial edema and intense mononuclear cell infiltration, composed of CD4+ T cells (73.4%), in the upper dermis (Fig. **3A-C**). Other cell sources included CD8+ T cells (12%) and CD68+ cells (38%), but neither CD20+ nor CD56+ cells were found. These results suggest the predominant involvement of antigen-presenting cells and antigen–responsive T cells at the self-saliva pricked skin in BD patients.

## DISCUSSION

There are several lines of evidence about the eiopathogenesis of oral disorders, such as BD, RA, and HSV infection, being considerable similar clinicopathology of aphthous ulceration. Classical skin pathergy test with a dull and thick needle (approximately 20 G) may often cause a pustule formation within 24 hours, and it has long been considered an important diagnostic reaction for BD [18-20]. The pathophysiological mechanism of this action has yet to be enigmatic, because the principal histology contains uncharacteristic inflammation; for example, variable degrees of vascular reaction, albeit absence of typical vasculitis, and non-specific perivascular inflammatory infiltrates in the dermis [23-29]. The sensitivity of skin pathergy in Japanese BD patients may be relatively lower than those in other races, such as Mediterranean districts (30-40%) [19]. The sensitivity has gradually been decreasing from ~70% to ~40% in some other countries (1970s *vs* 1998-2007, respectively) [25, 27, 30]. It is therefore unlikely that the diagnostic accuracy and its potential benefit of the pathergy reaction remain constant. Although little is known about the chronological decrease of the pathergy sensitivity, one may postulate the considerable alteration of patients’ life style, diet, and sanitary condition. Particularly, dietary habits have completely been changed in Japan, and in parallel with this, the prevalence of Japanese BD patients has gradually decreased in recent decades [31]. Apart from these environmental origins, a convincing evidence for the pathergy reaction has emerged that the povidone iodine sterilization preceded for skin surgery efficiently reduced the positive rate of pathergy reaction [28]. This finding is in agreement with our observation that the skin prick test was positive with neat saliva, but was negative with FS-saliva. Overall, these evidence series strongly suggest the involvement of intra-oral microorganism(s) in the pathergy reaction.

In our study, patients with most BD patients (9/10, 90%) demonstrated a positive skin prick specific for their own intra-oral microorganism(s). The similar trend in the skin prick was also found in substantial numbers of RA patients (3/5, 60%). Moreover, the prick reactivity can represent the relevant disease activity; that is, the tiny pustule appeared in patients with active oral ulceration, as was our recent investigation [22]. None of other controls (3 herpetic aphthous ulceration, 2 BD-unrelated EN, and 6 healthy subjects) had positive reactions by the self-saliva skin prick, indicating the highly specificity of the skin prick in BD and RA. Streptococcal species normally persists in the oral micro-flora, and more specifically, *Streptococcus (S) sanguinis* and *S. mitis* were increased significantly in saliva from BD patients [13, 14]. Combining with data demonstrating a hypersensitivity to oral streptococci in RA patients [17] and our finding that saliva culture from representative BD patients revealed streptococcal colonies, the positive skin prick reaction may be a consequence of the local hypersensitivity to streptococcal antigen(s). This is also supported by the histological similarity between oral aphthous lesion in BD/AU and skin pathergy reaction, exhibiting perivascular lympho-histiocytic infiltration in the dermis [15, 32, 33]. Immunohistologically, the infiltrated mononuclear cells were mainly composed of CD4+T cells and CD68+ monocyte/macrophages at the pricked skin sites, suggesting a DTH reaction. Moreover, the clinicopathology of these skin pathergy was inhibited by a low dose of systemic corticosteroid therapy just before the self-saliva skin prick, but it flared up by repeating the skin prick after cessation of the corticosteroid (data not shown). An extrapolation simply from a series of these evidences brings the scenario that accidental minor trauma and/or repeated irritation – even toothbrushing - in the oral mucous membrane may allow certain streptococcal species penetrate into the underneath oral tissue. The streptococcal antigen(s) can be processed to recognize properly with intra-epidermal immunocompetent cells, such as Langerhans cells, because the tip of the “Prick-Lancetter” used in this study never reaches to the dermis when it routinely stunk on the skin surface. Afterwards, the processed antigen-responsive helper T cells might be induced, then access to the prime-immunized oral mucosa, and finally establish aphthous ulceration in BD and RA [10, 15-17].

It remains unclear whether the genetic predisposition such as HLA-B51 association is correlated with streptococcal hypersensitivity in the clinicopathology of BD, but it is of interest that the production of IL-12 from peripheral blood mononuclear cells (PBMCs) stimulated by streptococcal antigen was rather stronger in BD patients without HLA-B51 than those with HLA-B51 [33, 34]. IL-12 can potentially induce a DTH reaction and has some biological effects as an initial inflammatory accelerator in BD [35]. The serologically uncommon KTH-1 type of *S. sanguinis* is increased significantly in the oral bacterial flora of BD patients compared with healthy controls [12-14]. In addition, circulating IgG antibodies against *S. sanguinis* are highly detectable in sera from BD patients, and these antibodies harbor a cross immunoreactivity with synthetic oligopeptides of human 65k-Da heat shock protein (HSP-65), a highly homologue of streptococcal HSP-60 [36, 37]. It is thus conceivable that BD patients have an acquired cross immunoreactivity to the similar sequences between streptococcal and human HSP counterpart molecules. From the species-specific point of view, a mouse model study has also demonstrated that part of clinical symptoms for BD is capable of recapitulating by inoculation of *S. sanguinis* from oral cavity in BD patients [38]. This suggests that the local immunization with *S. sanguinis* *via *oral mucous membrane can elicit BD-like symptoms over the certain species. BD and RA patients who spontaneously carry *streptococci* in their oral cavity might share the similar immune responses to their resident streptococci. A direct linkage between BD and streptococcal hypersensitivity is that *Bes-1* DNA encoding partial *S. sanguinis* genomic sequences was detectable in perivascularly infiltrated mononuclear cells in the oral/genital ulcerations and EN-like lesions of BD patients [39].

The lesional hypersensitivity to streptococcal antigens may lead to variable degrees of the downstream immune responses, because a baseline production of proinflammatory cytokines, INF-γ, IL-6, IL-8, and IL-12, was increased in PBMCs stimulated by *S. sanguinis* (KTH-1 strain) antigens [33, 40, 41]. For microbacterial antigen recognition, ten distinct Toll-like receptor (TLR) family members act as innate immune receptors against bacteria, viruses, and fungi [42]. Although TLRs are weakly detectable in various human tissues, the steady-state levels of the expression is much higher in the oral mucosa and digestive tracts, both of which are highly accessible with exogenous antigens [43]. The expression of TLR-3, -4 and -6 was enhanced in neutrophils and monocytes of BD patients when stimulated with HSP-65/60 and *S. sanguinis *antigens [44, 45]. The innate immune system may thus contribute to the acquisition of aberrant immune response against oral *streptococci* in the underlying immunopathogenesis of BD. On this basis, antibiotic therapy targeting oral streptococci and inducible immune tolerance using HSP-encoded oligopeptides can be therapeutic challenge, to inhibit the increased oral streptococci and the subsequent activation of cytokine cascades *via *streptococcal antigen-driven T cells [10, 46, 47].

In summary, the skin prick test using self-saliva (a new diagnostic pathergy) can be a safe and useful *in vivo* approach for differentiating BD and RA patients, particularly those who have active oral aphthous ulceration, from other mimicking oral aphthoses. The reaction is mediated by a hypersensitivity to Streptococci in oral resident micro-flora. We now warrant further evaluation for the acquired immune response specific for the common bacteria in some races, other than Japanese, and more importantly, need to address the mechanism detail for the establishment of such a primitive autoimmunity in BD and RA.

## Figures and Tables

**Table 1:** Skin Prick Test with Self-Saliva in 26 Subjects

No.	Subjects	Age/Sex	Self-Saliva		FS-Saliva	Control Saline
			Erythema (mm)	Pustules		
1	Neuro BD	55/M	11 x 15	+	nd	-
2	Incomplete	33/F	22 x 22	-	-	-
3		26/F	10 x 10	+	+ dot	-
4		27/M	11 x 12	+	+ dot	-
5		47/M	10 x 13	+	+ dot	-
6		36/F	5 x 10	-	nd	-
7		46/M	10 x 10	-	-	-
8		17/F	5 x 5	-	-	-
9		34/F	6 x 14	-	-	-
10	Complete	23/M	10 x 10	+	-	-
**Recurrent Aphthosis (RA)**						
11	24/F		8 x 10	-	-	-
12	28/F		8 x 4	-	-	-
13	32/F	-		-	nd	-
14	29/M	-		-	nd	-
15	28/F		3 x 5	-	-	-
**Disease Controls**						
16	EN	39/F	-	-	nd	-
17		61/F	-	-	-	-
18-20	3 Viral aphthosis		-	-	-	nd
21-26	6 Healthy controls		-	-	-	-

FS, filter-sterilized; BD, Behcet’s disease; EN, erythema nodosum; nd, not determined.
